# Editorial: Biomechanics and oxidative stress

**DOI:** 10.3389/fbioe.2022.933926

**Published:** 2022-08-15

**Authors:** Rita Payan-Carreira, Dario Santos, Subramani Kanagaraj, José António Simões

**Affiliations:** ^1^ Comprehensive Health Research Centre (CHRC), Department of Veterinary Medicine, Science and Technology School, University of Évora, Évora, Portugal; ^2^ Centre for the Research and Technology of Agro-Environmental and Biological Sciences, Department of Biology and Environment, University of Trás-os-Montes and Alto Douro, Vila Real, Portugal; ^3^ Centre for Sustainable Polymers, Indian Institute of Technology Guwahati, Guwahati, India; ^4^ College of Art and Design [ESAD], Matosinhos, Portugal

**Keywords:** biomaterials, oxidative stress, tissue reaction, healing, therapeutic approaches

Biomaterials have been used to stimulate the repair and regeneration of damaged tissue and replace lost or irrevocably non-functional tissues or organs. The clinical success of implants and their longevity depends on the interplay between the biomaterial, its structural design, and its physical and chemical characteristics and the local microenvironment. Mechanical forces at the implant site determine tissue healing and functional recovery. Ultimately, the material used in tissue repair and its mechanical behavior will determine its fate in the body, the local tissue acceptance, and its reaction to external loads. Oxidative stress is a major determinant in the interaction of biomaterials with the local tissue and ultimately a determinant of the success and bioadaptation of the implant.

Oxidative stress naturally occurs in living organisms. It is usually balanced and actively participates in multiple physiological functions. Nevertheless, when unbalanced, it leads to the accumulation of reactive oxygen species (ROS) and reactive nitrogen species (RNS), which are deleterious and trigger significant cell damage. While increased levels of reactive oxygen and nitrogen species have been consistently linked to disease processes, their presence is essential for the modulation of redox-sensitive pathways. Those pathways, which are partially characterized, are determinants for cell differentiation and fate and fundamental in response to stimuli. ROS has been shown to participate in immune cell recruitment and, thus, in tissue inflammation and healing processes. Even though it is now widely recognized that redox reactions are paramount in determining implant success and that oxidative/nitrosative stress (OS/NS) is a critical player in biomaterial behavior after implantation, the impact of oxidative stress on cellular biomechanics is not adequately understood. Because excessive OS/NS or inflammation at the site of implant application may compromise healing and tissue regeneration, positive modulation of antioxidant mechanisms may emerge as a powerful therapeutic approach.

Based on these premises, this Research Topic aims to collect new evidence on the relationship between biomaterials and implants and the local oxidative stress pathways, explore the effects of their physicochemical properties on the tissue biomechanics, and unveil molecular pathways behind the local tissue reaction against biomaterials and how these responses affect the implants bioadaptation. The manuscripts gathered under this collection address one or multiple aspects of the role of oxidative stress, tissue mechanical properties, and implant integration.

Disruption in lung extracellular matrix homeostasis and tissue changes in tissue stiffness and function is one of the most frequent progressive lesions described in amyotrophic lateral sclerosis (ALS). Using a mutated SOD1G93A rat model with impaired antioxidant capacity, Aydemir et al. showed that the disruption of the redox state originated significant biochemical and biomechanical changes in the lung leading to increased lung stiffness. This loss of function was associated with increased oxidative stress, decreased antioxidant capacity, altered trace element balance, and accumulation of mutated SOD protein (i.e., SOD1G93A). This study suggests that controlling SOD1 changes in the early stages of the disease may open new therapeutic approaches to ameliorate the well-being of ALS patients.

The importance of the integrity of antioxidant mechanisms in disease and as factors contributing to a return to homeostasis is illustrated by the experimental study by Li et al. Using a Wistar rat model, the authors detail the effects of inhaling ultrafine zinc particles on improving cardiac function and peripheral cardiovascular hemodynamics and the zinc acts by restoring the NF-κB and PPAR pathways and mitigating the deleterious side effects of oxygen radicals’ accumulation.

In their review, Reis and Ramos addressed the role of oxidative stress in bone. The authors stressed the importance of the redox balance in bone healing and remodeling processes and reviewed how an altered biomechanical environment is associated with increased ROS levels and inflammation, even though much is yet to uncover on how to fine-tune the mechanical stimuli promoting a beneficial equilibrium between bone resorption and formation.

Redox imbalance and implant failure may result from either a mismatched elastic modulus between the implants and the local tissue or an implant surface averse to proper target cell adhesion, differentiation, and proliferation. Aydemir et al.’s study highlighted the importance of the implant choice on the redox state and the success of acellular dermal matrix grafts for rotator cuff tear treatment and restoration of the glenohumeral joint biomechanics. This study hinted at the need to resource for autologous tissue to guarantee a reliable recovery of joint function.

Those results echo the findings reviewed by Luo et al. on hydrogel stiffness effect on the promotion of tissue repair. Hydrogel stiffness regulates the mesenchymal stem cell differentiation by ROS-mediated pathways and antioxidant capacity. The authors discuss how changes in hydrogel stiffness due to altered physical and chemical properties affect the interaction with surrounding cells and foster tissue repair by modulating local oxidative stress.

Taken together, the contributions to the “Biomechanics and Oxidative Stress” Research Topic elucidate key interactions between biomaterials, their biomechanical environment, and oxidative stress and open new avenues to different therapeutic approaches using biomaterials and implants.

